# Hermetic Seal

**Published:** 1867-12

**Authors:** 


HERMETIC SEAL.—A mixture of gelatine and glycerine is liquid while hot, but on cooling it becomes solid, retaining considerable elasticity and toughness. The neck of a bottle dipped into this melted compound is covered with an air-tight cap, which can be made as thick as desired by repeating the operation.

